# Parasitoid Causes Cascading Effects on Plant-Induced Defenses Mediated Through the Gut Bacteria of Host Caterpillars

**DOI:** 10.3389/fmicb.2021.708990

**Published:** 2021-09-06

**Authors:** Jie Wang, Charles J. Mason, Xueyang Ju, Rongrong Xue, Lu Tong, Michelle Peiffer, Yuanyuan Song, Rensen Zeng, Gary W. Felton

**Affiliations:** ^1^Key Laboratory of Ministry of Education for Genetics, Breeding and Multiple Utilization of Crops, College of Agriculture, Fujian Agriculture and Forestry University, Fuzhou, China; ^2^Department of Entomology, Pennsylvania State University, University Park, PA, United States; ^3^State Key Laboratory of Ecological Pest Control for Fujian and Taiwan Crops, Fujian Agriculture and Forestry University, Fuzhou, China

**Keywords:** maize, fall armyworm, parasitoid, gut bacteria, plant defense

## Abstract

Koinobiont endoparasitoid wasps whose larvae develop inside a host insect alter several important facets of host physiology, potentially causing cascading effects across multiple trophic levels. For instance, the hijacking of the host immune responses may have effects on how insects interact with host plants and microbial associates. However, the parasitoid regulation of insect–plant–microbiome interactions is still understudied. In this study, we used the fall armyworm (FAW), *Spodoptera frugiperda*, and the braconid parasitoid *Cotesia marginiventris* to evaluate impacts of parasitism on the gut microbiome of FAW larvae, and respective maize plant defense responses. The level of reactive oxygen species and the microbial community in larval gut underwent significant changes in response to parasitism, leading to a significant reduction of *Enterococcus*, while elevating the relative abundance of *Pseudomonas*. FAW with parasitism had lower glucose oxidase (GOX) activity in salivary glands and triggered lower defense responses in maize plants. These changes corresponded to effects on plants, as *Pseudomonas* inoculated larvae had lower activity of salivary GOX and triggered lower defense responses in maize plants. Our results demonstrated that parasitism had cascading effects on microbial associates across trophic levels and also highlighted that insect gut bacteria may contribute to complex interrelationships among parasitoids, herbivores, and plants.

## Introduction

Under field conditions, insects are exposed to a complex of parasites that lead to complicated interrelations, with parasitoid wasps acting as important top-down pressures and biological controls of agricultural pest (Pennacchio and Strand, 2006). The oviposition by parasitoids such as braconid and ichneumonid wasps transforms host physiology *via* the injection of a cocktail of egg, venom, and polydnaviruses (Asgari, 2006). To ensure parasitoid offspring development, these individual and collective agents of the parasitoids regulate the host through intertwined processes, including the suppression of immune defenses, inhibition of growth, and disruption of metamorphosis (Hassell and Waage, 1984; Dover et al., 1987; Strand and Pech, 1995; Pruijssers et al., 2009; Burke and Strand, 2012). Reactive oxygen species (ROS) is a major immune mechanism regulating insect gut microbiota homeostasis (Oliveira et al., 2011). In addition, ROS in *Drosophila* was found to play an important role in conferring resistance to wasp parasitism (Louradour et al., 2017). As a consequence of the disruption in immune functioning, the host microbiota is likely to be influenced after the parasitism. For example, injection of the venom from the parasitoid *Habrobracon hebetor* led to a significant shift of predominant bacteria in the gut microbiota of *Galleria mellonella* host larvae (Polenogova et al., 2019). In herbivorous insects, gut microorganisms can facilitate multiple roles mediating interactions across trophic levels (Engel and Moran, 2013; Douglas, 2015). However, the impact of the microbial shift in the host gut after parasitism on multitrophic interactions is still unclear.

Plants are able to perceive herbivore-associated cues that may be present in oral secretions (OS), oviposition secretions, herbivore feces, and others (Felton and Tumlinson, 2008; Zhu et al., 2014; Acevedo et al., 2015). Several studies showed that parasitoids could indirectly influence plant defenses through changing OS of host caterpillars. For example, *Microplitis croceipes*-parasitized corn earworm had lower glucose oxidase (GOX) activities in their saliva, which significantly downregulated plant defense responses (Tan et al., 2018). Moreover, the color of oral regurgitant of *Pieris rapae* and *P*. *brassicae* parasitized by *Cotesia glomerata* and *Hyposoter ebeninus* was found to be lighter and induced stronger defensive responses in cabbage plants (Poelman et al., 2011). Evidence from several studies shows that there are copious bacteria in larval OS or regurgitant arising from the anterior gut, which are the important sources of bacteria (Cardoza et al., 2006; Chung et al., 2013; Acevedo et al., 2017a; Shikano et al., 2017; Wang et al., 2017). Colorado potato beetle larvae were found to secrete bacteria during feeding and inhibit the jasmonic acid (JA)-regulated anti-herbivore defenses, but increased salicylic acid (SA)-regulated responses in tomato (Chung et al., 2013). Thus, insect symbiotic bacteria could play as a decoy to make plants incorrectly perceive the threat from herbivore insects as a microbial attack, resulting in lower plant defenses and enhanced larval growth.

*Cotesia marginiventris* is a generalist larval parasitoid that attacks many different lepidopterous species, including fall armyworm (*Spodoptera frugiperda*; FAW) (Turlings et al., 1991). We hypothesized that parasitism may influence the gut bacterial community of host caterpillar and thereby indirectly mediate plant defenses. We also suspected that different FAW resident bacterial isolates may exert distinct effects on confining the tritrophic interaction of parasitoid–insect–plant. To address these hypotheses, we firstly measured the effects of parasitism on fall armyworm gut physiology, bacterial communities, and salivary enzyme activities. Then, we studied plant defensive responses to attacks from the parasitized larvae. Finally, we reintroduced the predominant cultivable bacteria to axenic fall armyworm larvae to investigate how the gut bacteria influenced larval performance and plant defensive responses. These results will provide better understanding of the effect of the ecological role of the gut bacteria of lepidopterans in mediating multitrophic interaction among the parasitoid, insect, and plant.

## Materials and Methods

### Experimental Organisms

#### Plants

Seeds of the maize cultivar (*Zea mays*) inbred line B73 were kindly provided by the USDA-ARS. Maize seeds were germinated in Promix Soil Medium (Sunshine Mix4 Aggregate Plus, Sun Gro Horticulture) in a greenhouse (16L: 8D) at Pennsylvania State University, University Park, PA, United States. Ten days after germination, seedlings were transplanted into a 2:1 ratio of sterilized field soil and promix media and fertilized once with 3 g of Osmocote Plus (15-9-12, Scotts, 3 Marysville, OH, United States). Plants in the V5–V6 physiological stage were used for experiments.

#### Caterpillars

Fall armyworm (*S*. *frugiperda*, FAW) eggs were purchased from Benzon Research (Carlisle, PA, United States) and maintained in laboratory conditions. FAW larvae fed on maize leaves (cv. B73) were used for experiments unless otherwise noted.

#### Parasitic Wasps

*Cotesia marginiventris* is a generalist parasitoid for noctuid caterpillars, and we used FAW larvae to maintain *C*. *marginiventris* colony for multiple generations as previously described (Jones et al., 2020). Adult wasps were sexed using the length of antenna and presence of an ovipositor (Lewis and Burton, 1970). Parasitized caterpillars in all experiments were used 5 days after parasitism (the second day of fourth instar stage) unless noted otherwise, and non-parasitized caterpillars at the same developmental stage were used as controls. All insect colonies were maintained in a growth incubator with a 16-h photoperiod at 25°C.

### Insect Physiological Responses to Parasitism

#### Effects of Parasitism on ROS Levels in Larval Gut

Reactive oxygen species play an important role in insect gut immunity (Bae et al., 2010), and hydrogen peroxide (H_2_O_2_) belonging to ROS is an important indicator of oxidative stress and immune responses (Bi and Felton, 1995). To examine if parasitism could affect the ROS level in larval guts, the ferrous oxidation-xylenol orange (FOX) assay was modified and used for comparative spectrometric analysis of H_2_O_2_ levels in the gut of parasitized and non-parasitized FAW larvae. H_2_O_2_ causes the oxidation of ferrous ion to ferric ion, and the complex formation of the reduced ion with xylenol orange produces a blue chromosphere which is detectable at 560 nm (Jiang et al., 1992; Nappi and Vass, 1998). Briefly, gut tissues were weighed and homogenized in 200 μl PBS (0.1 M, pH = 7.0). For reaction, 50 μl of homogenate was collected in a sterile tube containing 450 μl of a FOX working reagent that was composed of ammonium ferrous sulfate, H_2_SO_4_, reagent-grade methanol with 4 mM butylated hydroxytoluene (BHT), and xylenol orange in Milli-Q H_2_O. After a 30-min incubation at room temperature, 200 μl was added to duplicate wells in a 96-well plate. The absorbance of each sample was read at 560 nm. Standard curves were conducted using commercial hydrogen peroxide.

#### Effect of Parasitism on Bacterial Load in Larval Gut and Oral Secretions

Reactive oxygen species plays a pivotal role in regulating the composition of the insect gut bacterial community (Diaz-Albiter et al., 2012; Xiao et al., 2017). A recent study shows that parasitoid envenomation alters the immunity and gut microbiota of the *G*. *mellonella* (Polenogova et al., 2019). To test if this phenomenon also occurs in the system of *C*. *marginiventris* and FAW larvae, we measured the number of bacteria in the gut of larvae with or without parasitism. Five days post parasitism, caterpillars were surface sterilized in 70% ethanol and washed twice with sterilized water. The midguts and contents were isolated, weighed, and homogenized in 1 ml of 0.1 M PBS. Lepidopteran larvae can produce OS arising from their gut during their feeding (Peiffer and Felton, 2009; Acevedo et al., 2015, 2017a). Thus, we also quantified the bacterial load in the OS of larvae with or without parasitism to verify that bacterial load in OS was consistent with that of the larval gut. Two microliters of crude OS was freshly collected from the oral cavity of caterpillars by gently tapping their heads according to a previously described procedure (Peiffer and Felton, 2009; Wang et al., 2017). Then, the suspension was diluted with 0.1 M PBS to 10^–2^, 10^–3^, 10^–4^, and 10^–5^, and 100 μl of each aliquot was added to 2YT agar media to count numbers of colonies (colony forming units, CFU). The petri dishes were incubated at 27°C.

#### DNA Extraction and Illumina Sequencing of the Midguts of Caterpillars With Parasitism

Midgut tissues from parasitized and non-parasitized caterpillars were collected under sterile conditions and stored at –80°C until DNA extraction. DNA extractions were conducted using the Quick-DNA^TM^ Fecal/Soil Microbe Microprep Kit (Zymo Research, Irvine, CA, United States) according to the instructions from the manufacturer. The V4 region of the 16S rRNA gene was analyzed using the Illumina MiSeq PE300 sequencing platform at the PSU Hershey Genomics Facility (Penn State University, PA, United States). The primers used for amplification were 515F and 806R (Caporaso et al., 2012). Amplicons were generated in 25-μl volumes using Phusion Hi-Fidelity Polymerase (New England Biolabs, Ipswich, MA, United States) containing 0.5 μM of forward and reverse primers and 25 ng of template DNA. Reaction conditions for 16S amplification were 94°C 3 min, 30 cycles of 94°C for 45 s, 50°C for 60 s, and 72°C for 90 s, followed by a final extension of 72°C for 10 min.

The generated paired-end reads were managed using FLASH software (V1.2.7) by setting to the minimum overlap of 10 bp with other default parameters (Magoč and Salzberg, 2011). The dataset was analyzed using Quantitative Insights into Microbial Ecology (QIIME, V1.7.0) (Caporaso et al., 2010). Aligned sequences were selected within a size range of 250–350 bp with less than 10 homopolymers and any ambiguous position. All sequences that did not align to the Silva 132 database were filtered out. Chimeric sequences were initially identified by the UCHIME algorithm in USEARCH with the script *identify_chimeric_seqs*.*py* implemented in the QIIME software. The script *filter_fasta*.*py* was used for removing chimeric sequences, and then sequences were clustered into operational taxonomic units (OTUs) by USEARCH with the script *pick_otus*.*py* at a similarity level of 97% (Edgar, 2010). The representative OTUs were selected based on the most abundant sequence in each OTU, and then the ribosomal database project (RDP) classifier tool was used to classify all sequences into different taxonomic groups (Wang et al., 2007). As there are abundant chloroplasts of maize leaves, the sequences that were classified as chloroplast were removed. The rarefaction, richness estimators, and diversity indices were estimated using QIIME software (Colwell and Coddington, 1994; Cole et al., 2009). The rarefied OTU table was generated using the script *multiple_rarefactions*.*py* and used for calculating alpha diversity indices by the script *alpha_diversity*.*py*, and the results were concatenated into a single file by the script *collate_alpha*.*py*. For beta diversity analysis, sequences were normalized based on the Bray–Curtis similarity matrix. Nonmetric multidimensional scaling (NMDS), analysis of similarities (ANOSIM), constrained principal coordinate analysis (PCoA), and permutational analysis of variance (PERMANOVA) were carried out using R (version: 3.4.3) as detailed in the Supplementary Information (Chen et al., 2018; Oksanen et al., 2018). Raw sequencing data were deposited in the NCBI SRA under BioProject PRJNA681731.

#### Effects of Insect Parasitism on the Interaction With Host Plant

##### Plant defense responses to the feeding of parasitized caterpillars

Herbivore cues of FAW caterpillars are known to induce production of proteinase inhibitors in maize (Chuang et al., 2014). To determine if parasitism by *C*. *marginiventris* influences the ability of FAW larvae to mediate plant defenses, maize plants were separated into three groups: parasitized caterpillar feeding, non-parasitized caterpillar feeding, and control plants (CK) without damage. Each caterpillar was placed in a clip cage on the third (from the top) terminal leaflet of each maize plant, which restricts caterpillars to consuming a similar amount of leaf tissues. The cages and caterpillars were removed after 10 h, and only plants consumed by caterpillars with the entire caged area (ca. 3.15 cm^2^) during the prescribed time period were selected for the following experiments of measuring plant defense responses. Twenty-four hours after placing the caterpillars on the plants, 50 mg of leaf around the feeding sites was collected for measuring the activity of trypsin protease inhibitors (trypsin PI), which could suppress the activity of digestive serine proteases in insects impairing their development (McManus and Burgess, 1995). Another 100 mg of leaf was harvested for measuring the relative expression of the maize proteinase inhibitor (*MPI*) gene, which was a wounding or insect feeding-inducible gene in plants (Tamayo et al., 2000). Leaf samples were frozen with liquid nitrogen and stored at –80°C before analysis.

The activity of trypsin PI was measured as described previously (Chung and Felton, 2011). Briefly, leaf samples were powered by the Genogrinder (Geno/Grinder 2000, SPEX SamplePrep) and extracted with 1.25 ml buffer (0.046 M *Tris* buffer, pH 8.1, 0.0115 M CaCl_2_) containing 5% (w/v) insoluble polyvinylpolypyrrolidone. Samples were centrifuged at 4°C, 11,000 × *g* for 10 min, and the supernatant was collected for the measurement. Twenty microliters of the supernatant was mixed with 10 μl of 1 mM HCl containing 0.4 μg bovine trypsin (Sigma, St Louis, MO, United States; T1426) and 70 μl of extraction buffer. The mixture was incubated for 10 min at room temperature, mixed with 100 μl of 0.002 M *p*-toluenesulfonyl-L-arginine methyl ester (Sigma T4626), and monitored for 5 min at 247 nm. The trypsin PI activity was calculated as PI(%) = (1 − (*A*/*C*)) × 100, where *A* represents trypsin activity of the sample and *C* represents the maximum value of trypsin activity in CK.

RNA was extracted with TRIzol reagent (Ambion). One microgram of RNA was used to synthesize cDNA with the High-Capacity cDNA Reverse Transcription Kit (Applied Biosystems) in a PCR thermal cycler (GeneAmp PCR System 9700). The *MPI* gene has been widely used as a marker to evaluate herbivore-induced defense responses in maize plants (Tamayo et al., 2000; Ray et al., 2015; Acevedo et al., 2017a). Primers used for quantitative real-time PCR (qRT-PCR) analysis are listed in Supplementary Table 1. qRT-PCR analysis was performed by QuantStudio 3 (Thermo Fisher, United States) with SYBR Green PCR Mix (Applied Biosystems). The relative expression of target genes was compared with intact control groups by using the 2^–ΔΔct^ method (Livak and Schmittgen, 2001).

##### Host larvae performance on plants damaged by parasitized larvae

To examine the effect of plant-induced defense on parasitized caterpillar performance, we conducted a bioassay by analyzing the larval relative growth rate (RGR). Plants were damaged by larvae with or without parasitism that were confined to a leaflet as described above, and the damaged leaflets were detached after 24 h. At the last day of the second instar stage, FAW larvae of similar body size were selected and parasitized by *C*. *marginiventris*. Caterpillars were weighed and then placed on excised maize leaves in a 1-oz cup containing 2% agar to keep leaves moist. Three days later, caterpillars were reweighed and RGR was calculated using the equation: (*end weight* − *start weight*)/(*average weight* × days) (Felton et al., 1989).

### Functional Validation of the Role of Gut Bacteria in the Response of Both Insect and Plant to Parasitism

#### Axenic Larvae Rearing

The axenic FAW colony was generated as previously described (Mason et al., 2019). Eggs of FAW were collected from mating containers and surface sterilized with 2.5% bleach before use in experiments. Larvae were hatched from surface-sterilized eggs in autoclaved containers and fed with sterile wheat germ diet until the end of second instar. The autoclaved wheat-germ-based diets included all components of the previously described diet (Chippendale, 1970) except bactericidal antibiotics, as the antibiotics may compromise establishment of bacteria in the following experiments (Mason et al., 2020). The detailed recipe of the wheat-germ-based diet used in the current study is described in the Supplementary Data Sheet 1. Feedings were conducted in an aseptic laminar flow hood. Individual larvae with or without bacterial inoculation were maintained in 22.5-ml plastic cups that were sterilized with 70% ethanol and placed under UV light for 20 min. Artificial diets were autoclaved and poured into sterile petri dishes. We then homogenized the entire caterpillar and verified the axenic state of insects through plate culturing by counting colony-forming units (CFUs).

### Effect of Bacterial Inoculation on Caterpillar Salivary Glucose Oxidase Activity

*Pseudomonas* sp. (FAW10-3A) and *Enterococcus* sp. (FAW13-5) were previously isolated from fall armyworm larvae (Jones et al., 2019). Before reintroducing bacteria back to axenic larvae, each isolate was grown individually in 2YT media (16 g tryptone, 10 g yeast extract, and 5 g NaCl dissolved in 1 l Milli-Q water) at 27°C overnight. The bacterial culture was centrifuged at 5,000 *g* for 10 min, and cells were resuspended in 10 mM MgCl_2_ solution (Chung et al., 2013). We used the sterile pinto bean diet instead of wheat germ diet to reintroduce bacteria to axenic larvae, because it has been reported that pinto bean diet was more suitable to establish bacteria in the larvae compared to wheat germ diet (Mason et al., 2020). Each group of insects at the second instar stage was fed twice with a small piece of pinto bean diet cube (c.a. 0.5 cm diameter) containing 10^7^ viable cells. The pinto bean diet was made of dried pinto bean powder, fortified yeast, ascorbic acid, methylparaben, sorbic acid, propionic acid, agar, and vitamin mix as previously described (Shorey and Hale, 1965). The detailed recipe of the pinto bean diet used in this study is described in the Supplementary Data Sheet 2. Control larvae received an identical volume of sterilized MgCl_2_ solution. After 5 days, the labial glands from caterpillars inoculated with or without bacteria were collected for GOX activity measurement. As previously described (Eichenseer et al., 1999), and the protein concentration was determined with a Bradford assay (Bradford, 1976).

### Impacts of Caterpillar Inoculated With Bacteria on Defense Responses in Maize Plants

Fall armyworm larvae with or without re-inoculation as mentioned above were assigned to feed on maize plants. These larvae were placed on plants using clip cages, as mentioned above. Each treatment had five replicates. After 24 h, the leaf tissue (50 mg) around the feeding or damaged sites was harvested for further measurement of trypsin PI activity and *MPI* gene expression.

### Statistical Analyses

The normal distribution and homogeneity of the data were verified before analysis. When needed, the variables were transformed to meet the assumptions of normality. Differences were considered as significant when *p* < 0.05. Larval physiological responses such as H_2_O_2_ quantification, CFU analysis with log_10_ transformation, GOX activities, and alpha-diversity indices were compared between parasitized larvae and non-parasitized larvae using unpaired Student’s *t*-test. Plant defense responses such as trypsin PI activity and the expression of *MPI* gene, caterpillar salivary GOX activities in response to bacterial inoculation, and caterpillar performance bioassay (RGR) were analyzed with one-way ANOVA following the Fisher’s test (α = 0.05). All data were analyzed using Minitab (Minitab Inc., State College, PA, United States), and figures were generated using GraphPad Prism 8 (GraphPad Software Inc., San Diego, CA, United States).

## Results

### Parasitism Affected the Level of H_2_O_2_ and Bacterial Load in Larval Guts

**FIGURE 1:**
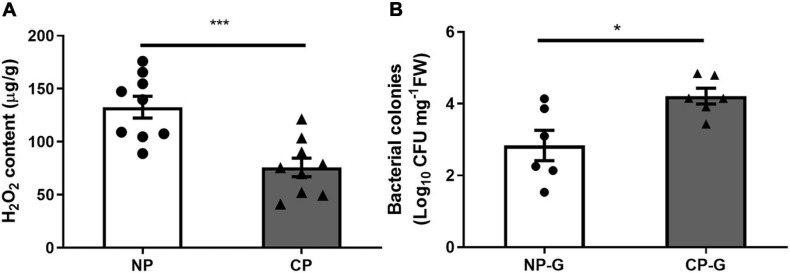
Parasitism inhibited hydrogen peroxide (H_2_O_2_) levels and increased the bacterial load in the gut of fall armyworm larvae. **(A)** H_2_O_2_ levels in the larval guts were measured using the FOX assay. NP, caterpillars without parasitism; CP, caterpillars parasitized by *C*. *marginiventris*. Values are untransformed mean ± SEM (*t* = 4.21, *p* < 0.001, *n* = 9; unpaired *t*-test). **(B)** The number of bacteria in the larval gut was quantified by analyzing colony forming units (CFU). NP-G, gut tissue of caterpillars without parasitism; CP-G, gut tissue of caterpillars parasitized by *C*. *marginiventris*. Values are mean ± SEM with log10 transformation (*t* = 2.894, *p* = 0.016, *n* = 6; unpaired *t*-test). The asterisk indicates significant differences between treatments (*p < 0.05; ***p < 0.001).

To determine if parasitism affects the levels of ROS, the H_2_O_2_ level in the larval gut was measured using FOX assay. Parasitism by *C*. *marginiventris* significantly inhibited the level of H_2_O_2_ in the gut compared to *S*. *frugiperda* larvae without parasitism (Figure 1A; *t* = 4.21, *p* < 0.001). By traditional culturing, the CFU number of bacteria in the gut of parasitized caterpillar was significantly higher than that of the un-parasitized group (Figure 1B; *t* = 5.307, *p* < 0.001). A similar result was also found in the oral secretion of fall armyworm larvae that arises from the gut, where the CFU number of bacteria was much higher in the oral secretion of parasitized larvae than that in non-parasitized larvae (Supplementary Figure 1; *t* = 2.384, *p* = 0.044). These results demonstrated that parasitism by *C*. *marginiventris* inhibited the ROS level and increased the bacterial load in the larval gut.

### Parasitism Caused Changes in Larval Gut Microbiome Composition

**FIGURE 2:**
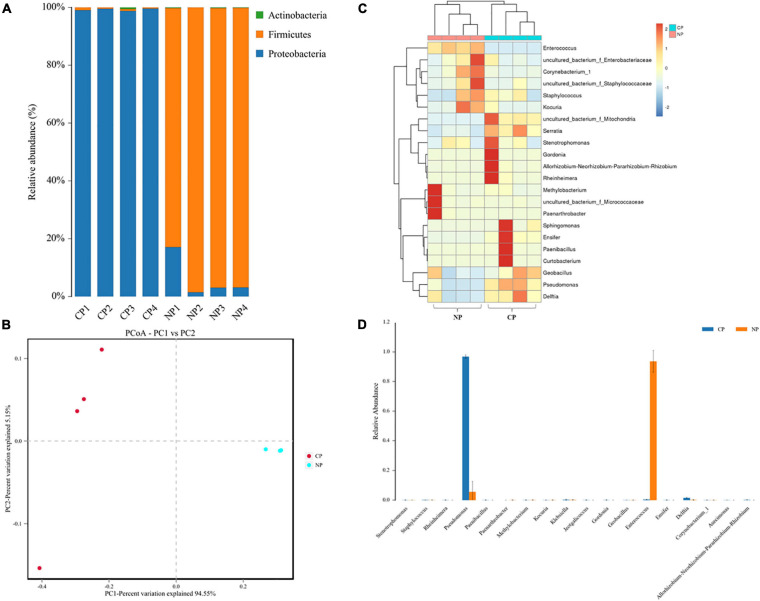
Parasitism alters the gut bacterial community composition. **(A)** The color-coded bar plot of the average bacterial phylum distributed between parasitized larvae and non-parasitized larvae. **(B)** Principal coordinate analysis (PCoA) of symbiotic bacterial communities in the gut of parasitized larvae and non-parasitized FAW larval plots based on Bray–Curtis metric (PERMANOVA test, *R*^2^ = 0.922, *p* = 0.03). **(C)** Heat map in log scale depicting the relative abundance of the top 20 shared genera and genus, with cluster analysis using the Bray–Curtis distance, followed by a complete-linkage method. **(D)** Relative abundance of bacteria at the genus level was compared between parasitized caterpillars and non-parasitized caterpillars using a nonparametric Wilcoxon rank-sum test. NP, caterpillars without parasitism; CP, caterpillars parasitized by *C*. *marginiventris*.

In the eight representative gut samples of FAW larvae with or without parasitism, a total of 511,199 clean reads were generated from both parasitized and un-parasitized caterpillars and grouped into 29 OTUs at the 97% similarity cutoff level, and the average number of OTUs identified from parasitized larva was higher than that of un-parasitized larvae (Supplementary Table 2 and Supplementary Data Sheet 3). Rarefaction curves indicated that the bacterial diversity of the individual sample was well covered by the sequencing analysis (Supplementary Figure 2). Venn diagrams showed that 20 OTUs were shared by two groups, which comprised 95.24 and 71.43% of the total OTUs of unparasitized larvae and parasitized larvae (Supplementary Figure 3). The above OTUs were assigned to the three main phyla Proteobacteria, Firmicutes, and Actinobacteria. The Firmicutes phylum in non-parasitized caterpillars had a higher percentage of the gut bacterial community, while parasitism shifted the dominant position of Firmicutes to Proteobacteria (Figure 2A). Alpha diversity was estimated by comparing the Chao1, ACE, Shannon, and Simpson indices (Table 1). The results indicated that the parasitized larvae had higher values for all the four indices than did the un-parasitized larvae, which suggested that parasitism with *C*. *marginiventris* caused a significant change in the gut bacterial community of *S*. *frugiperda* larvae. Principal coordinate analysis (PCoA) showed that the bacterial community structure in the gut of parasitized larvae significantly differed between larvae with parasitism and non-parasitized larvae (Figure 2B). In the scatter plot, PC1 and PC2 explained 94.55 and 5.15% of the data variation, respectively, clearly separating each group (PERMANOVA test with 999 permutations, *p* = 0.03). Similarly, the NMDS diagram indicated that bacterial communities of parasitized larval gut samples clustered independently and distinctly from non-parasitized larval gut samples using the Bray–Curtis similarity metric (Supplementary Figure 4, ANOSIM, *p* = 0.027). These results suggested that parasitism affected the evenness and structure of gut bacterial community compared to that of non-parasitized larvae.

**TABLE 1:** Comparison of alpha-diversity indices of gut bacterial community structure in fall armyworm larvae.

Index	NP	CP	*p*-value
			
Shannon	0.4047 ± 0.1352	1.278 ± 0.1193	0.0029**
Simpson	0.122 ± 0.0591	0.5138 ± 0.0509	0.0024**
ACE	14.5783 ± 1.5250	21.3044 ± 1.2526	0.0143*
Chao 1	14.125 ± 1.1968	20.875 ± 1.2311	0.0077**

*The diversity indices (Shannon, Simpson) and species richness indices (ACE, Chao1) were determined at 97% sequence similarity from each sample.*

*NP, caterpillars without parasitism; CP, caterpillars parasitized by Cotesia marginiventris.*

Values are untransformed mean ± SEM. The asterisk indicates significant differences between treatments (*p < 0.05; **p < 0.01).

The heat map of the OTUs assigned to the genus level provided a detailed view of the composition of the gut bacterial community between the two groups (Figure 2C). Remarkably, the bacterial community from *C*. *marginiventris* parasitized larvae contained a significantly higher proportion of *Pseudomonas* than those from non-parasitized larvae, while the proportion of *Enterococcus* was significantly lower in parasitized larvae than that in non-parasitized caterpillars (Figure 2D; Wilcoxon rank-sum test, *p* < 0.001). These results indicated that parasitism led to a shift in the dominant gut-associated bacteria of *S*. *frugiperda* larvae, and the dominant bacteria may have a negative effect on larval performance together with the effect of parasitism.

### Effects of Parasitism and Bacterial Inoculation on Salivary GOX Activities of Caterpillars

**FIGURE 3:**
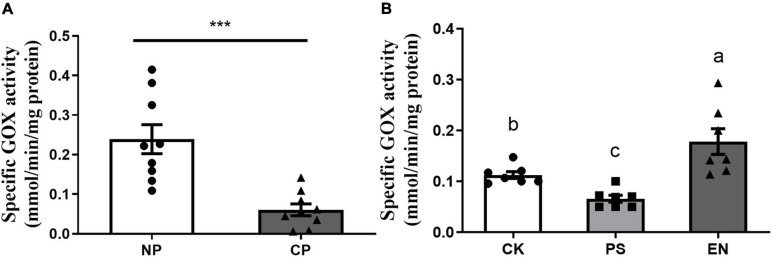
Effect of parasitism and bacterial inoculation on glucose oxidase activity in the labial glands of fall armyworm. **(A)** Glucose oxidase (GOX) activity in the labial glands of caterpillars was measured at day 5 after parasitism. NP, caterpillars without parasitism; CP, caterpillars parasitized by *C*. *marginiventris*. Values are untransformed mean ± SEM (*t* = 4.497; *p* = 0.0004; *n* = 9; unpaired *t*-test). The asterisk indicates significant differences between treatments (***p < 0.001). **(B)** GOX activity in the labial glands of fall armyworm with or without bacterial inoculation. Values are untransformed mean ± SEM (*F*_2_,*_18_* = 13.09, *p* < 0.001; *n* = 7, Fisher’s test). Different letters indicate significant differences between treatments.

Fall armyworm larvae parasitized by *C*. *marginiventris* showed significantly lower GOX activities in the labial glands than caterpillars without parasitism (Figure 3A, *t* = 4.497, *p* = 0.0004). The activity of GOX in salivary glands from caterpillars reintroduced with *Pseudomonas* (FAW10-3A) was significantly lower than the activity of caterpillar controls treated with MgCl_2_ buffer, while *Enterococcus* (FAW13-5)-inoculated larvae had significantly higher GOX activities in the labial glands compared to buffer-inoculated larvae (Figure 3B; *F*_2,18_ = 13.09, *p* < 0.001).

### Fall Armyworm Larvae With Parasitism Triggered Lower Defense Responses in Maize Plants

**FIGURE 4:**
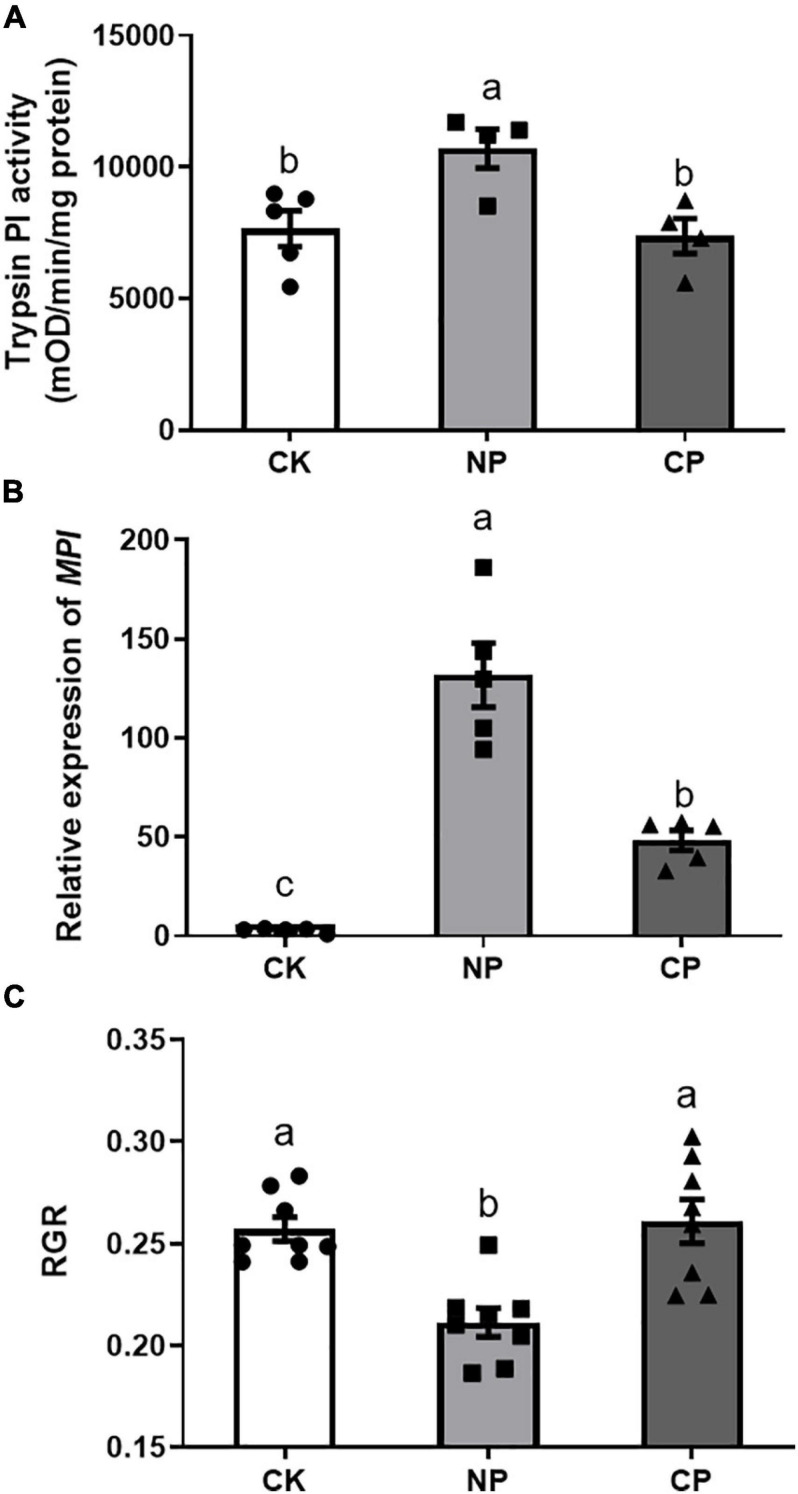
Effect of fall armyworm larvae with parasitism on inducing defense responses in maize. **(A)** Trypsin proteinase inhibitor (Trypsin PI) activity in maize plants (*F*_2,12_ = 6.703, *p* = 0.014; *n* = 5, Fisher’s test). **(B)** Maize proteinase inhibitor (*MPI*) gene expression in maize plants (*F*_2,12_ = 44.64, *p* < 0.001; *n* = 5, Fisher’s test). **(C)** Relative growth rate (RGR) of parasitized caterpillars feeding on plants previously treated with parasitized caterpillars (*F*_2,21_ = 11.52, *p* = 0.0004; *n* = 8, Fisher’s test). Values are untransformed mean ± SEM. Different letters indicate significant differences between treatments. CK, undamaged plants; NP, maize plants damaged by non-parasitized caterpillars; CP, maize plants damaged by caterpillars parasitized by *C*. *marginiventris*.

Larvae parasitized by *C*. *marginiventris* induced significantly lower levels of trypsin PI than did the larvae without parasitism (Figure 4A, *F*_2,12_ = 6.703, *p* = 0.014). In addition, we examined the transcript levels of the defense-related *MPI* gene-encoding protease inhibitor and found that larvae parasitized by *C*. *marginiventris* induced a lower expression of *MPI* than did non-parasitized larvae, respectively (Figure 4B; *F*_2,12_ = 44.64, *p* < 0.001). The RGR of parasitized larvae feeding on maize plants damaged by parasitized caterpillars was higher than that of caterpillars feeding on maize plants treated with non-parasitized caterpillars (Figure 4C, *F*_2,21_ = 11.52, *p* = 0.0004).

### Impacts of Caterpillars Inoculated With Bacteria on Plant Defenses

**FIGURE 5:**
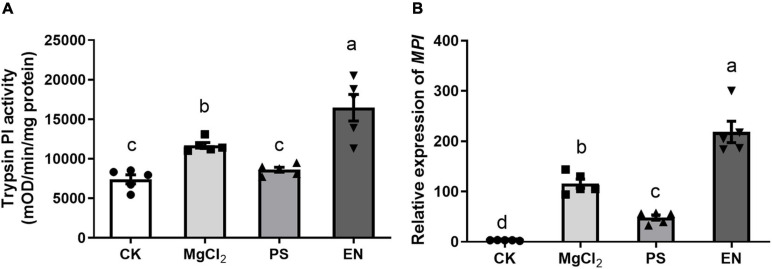
Impacts of caterpillars inoculated with *Pseudomonas* (PS) and *Enterococcus* (EN) on herbivore-induced defense response in maize plants. **(A)** Trypsin proteinase inhibitor (Trypsin PI) activity (*F*_3,16_ = 19.12, *p* < 0.001, Fisher’s test) and **(B)** *MPI* gene expression (*F*_3,16_ = 62.14, *p* < 0.001, Fisher’s test) in maize plants treated by fall armyworm larvae inoculated with PS or EN. CK, plants without any damage; MgCl_2_, plants damaged by caterpillars with only MgCl_2_ buffer. Values are untransformed mean ± SEM (*n* = 5). Different letters indicate significant differences between treatments.

We further tested if the presence of *Pseudomonas* FAW10-3A or *Enterococcus* (FAW13-5) would affect FAW larvae mediating defense responses in maize. Maize plants damaged by *Pseudomonas*-inoculated larvae had significantly lower trypsin PI activity, while *Enterococcus*-inoculated larvae triggered higher activity of trypsin PI in maize compared to plants damaged by buffer-inoculated caterpillars (Figure 5A; *F*_2,12_ = 25.46, *p* < 0.001). Similar results were also observed in *MPI* gene expression (Figure 5B; *F*_2,12_ = 87.90, *p* < 0.001). These results indicated that different bacterial strains may play a distinct role in regulating insect performance on plants.

## Discussion

Parasitoids remodel host physiology in a manner that cascades across multiple trophic interactions. Our study illuminates some of the complex multitrophic interactions elicited by parasitoids on caterpillar biology. We documented changes in host immune responses such as ROS in the gut, which may have led to a significant shift of the bacterial composition in the herbivore gut. Parasitism and symbiotic bacterial inoculation triggering changes in salivary GOX caused cascade effects on plant-induced defense. Studies on host–parasitoid interactions and on plant–herbivore interactions have largely developed independently, and our results suggest a greater need to integrate these fields with host insect symbiotic microbiota.

Oviposition by parasitoids such as braconid wasps could have a massive influence on host larval physiology (Fathpour and Dahlman, 1995; Hegazi et al., 2005; Bitra et al., 2011; Burke and Strand, 2012). Previous studies showed that ROS in the gut play an important role in defending against microbial infection (Ha et al., 2005a, b), and insects rely on the Duox-ROS system to mediate the proliferation of gut bacteria and maintain gut microbial homeostasis (Xiao et al., 2017). Therefore, a lower H_2_O_2_ level in the parasitized larvae may cause destabilization of normal-functioning host–microbe interactions. To be more specific, *Enterococcus* in the gut of FAW larvae was significantly decreased, while the relative abundance of *Pseudomonas* was significantly induced in *C*. *marginiventris*-parasitized larvae. Both *Enterococcus* and *Pseudomonas* have been frequently isolated from *S*. *frugiperda* (De Almeida et al., 2017; Jones et al., 2019; Gichuhi et al., 2020; Rozadilla et al., 2020; Ugwu et al., 2020). The gut bacterial diversity of *S*. *frugiperda* larvae also varied in samples from different environments, and the environmental condition could be an important driver of microbial community composition. For example, FAW larvae from the field population tended to have higher diversity of gut bacteria than those from the laboratory population (Jones et al., 2019). Low bacterial diversity in the gut of FAW larvae in the current study could be due to the laboratory rearing effects. The relatively sterile condition and lower diversity in foods may lead to lower diversity of gut bacteria in laboratory-maintained caterpillars (Xiang et al., 2006). Field-collected larvae should be included in future studies for comparatively analyzing the effects of parasitism on gut microbial communities.

The bottom-up effects of plant defense traits on parasitoids have been well appreciated (Kaplan et al., 2016; Hopkins et al., 2017), while the top-down effects of parasitoids on plant-induced defenses have just recently been receiving attention (Poelman et al., 2011; Tan et al., 2018; Volkoff et al., 2018; Zhu et al., 2018). Previous studies showed that the downregulation of GOX in *H*. *zea* parasitized by *M*. *croceipes* caused cascading effects across trophic levels and triggered a lower expression of plant defenses during caterpillar attacks (Tan et al., 2018, 2019). GOX is one of the most abundant proteins in the salivary secretions of FAW larvae and functions as an elicitor of maize defense expression (Chuang et al., 2014; Acevedo et al., 2017b). This study also confirmed that FAW larvae parasitized by *C*. *marginiventris* had lower levels of salivary GOX compared to that of non-parasitized larvae. Our previous study showed that insect gut-associated bacteria influenced the GOX activities in the salivary glands of corn earworm and indirectly mediated tomato-induced defenses (Wang et al., 2017). Insects harbor microbes in their digestive system that manipulate host physiology and shape insect–plant interactions (Oliver and Martinez, 2014; Hammer and Bowers, 2015). Here we report a similar result in FAW larvae parasitized by *C*. *marginiventris*, where parasitized caterpillars induced a lower expression of defense responses in maize. In addition, caterpillars inoculated with *Pseudomonas* and *Enterococcus* had distinct salivary GOX activities and therefore caused different effects on maize defense responses. The complex changes happening inside infected larvae may alter their ability to mediate plant defense responses. Thus, we predicted that the regulation of plant defense responses by caterpillar-associated microbes may be a widely occurring phenomenon influencing the top-down interactions.

Interactions among plants, insects, and predators can be very complicated, especially as insect gut microbiota is superimposed on these studies. Our findings revealed that changes in the gut microbes of the host larvae could be an important component in regulating top-down effects on insects and plants. Remarkably, the interactions of the caterpillar with its host plant may be mediated by parasitoids and gut bacteria. Further investigations are needed to understand the mechanisms through which parasitoids alter the host gut microbiota.

## Data Availability Statement

The datasets presented in this study can be found in online repositories. The names of the repository/repositories and accession number(s) can be found in the article/Supplementary Material.

## Author Contributions

JW and GF conceived the research. JW, CM, and MP conducted the experiments. JW, XJ, RX, and LT analyzed the data and conducted the statistical analyses. JW, CM, YS, RZ, and GF wrote and revised the manuscript. All authors read and approved the manuscript.

## Conflict of Interest

The authors declare that the research was conducted in the absence of any commercial or financial relationships that could be construed as a potential conflict of interest.

## Publisher’s Note

All claims expressed in this article are solely those of the authors and do not necessarily represent those of their affiliated organizations, or those of the publisher, the editors and the reviewers. Any product that may be evaluated in this article, or claim that may be made by its manufacturer, is not guaranteed or endorsed by the publisher.

## Supplementary Material

Supplementary Figure 1 Parasitism increased the bacteria load in the oral secretion of fall armyworm larvae.

Supplementary Figure 2 Rarefaction curves of larval gut samples.

Supplementary Figure 3 Venn diagram of the shared and unique OTUs between parasitized larvae and non-parasitized larvae.

Supplementary Figure 4 Non-metric multidimensional scaling (NMDS) analysis.

Supplementary Table 1 List of primers for quantitative real-time PCR for maize.

Supplementary Table 2 OTU and sequence number in individual sample.

Supplementary Data Sheet 1 R script for ANOSIM and PERMANOVA.

Supplementary Data Sheet 2 Preparation of both wheat germ diet and pinto bean diet.

Supplementary Data Sheet 3 Detailed information of OTU distribution and taxonmy in individual sample.
