# Supplementary figures and images for: Parasitoid Causes Cascading Effects on Plant-Induced Defenses Mediated Through the Gut Bacteria of Host Caterpillars
Source: Front Microbiol. 2021 Sep 6;12:708990. doi: 10.3389/fmicb.2021.708990 (PMC8452159; doi:10.3389/fmicb.2021.708990)

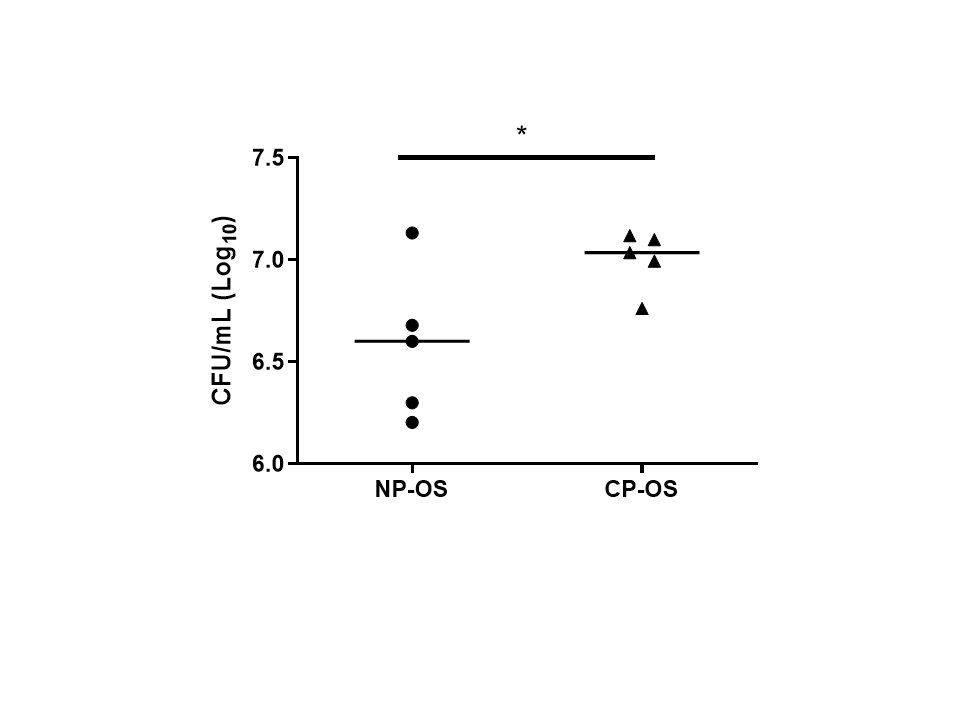

Supplement: Supplementary Figure 1 — Parasitism increased the bacteria load in the oral secretion of fall armyworm larvae. [file Image_1.tif]

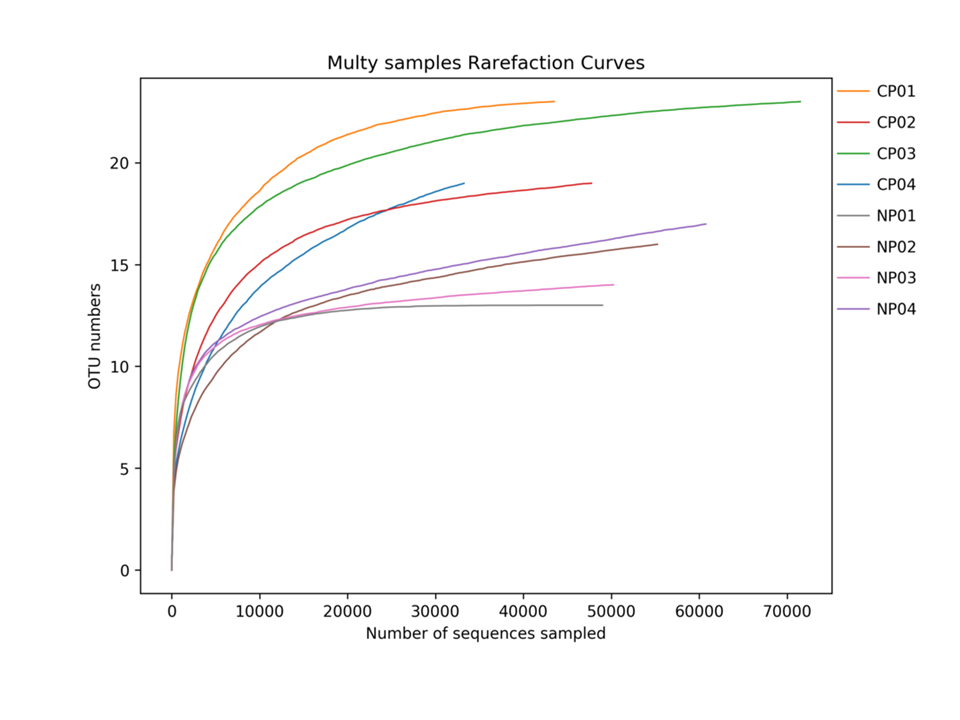

Supplement: Supplementary Figure 2 — Rarefaction curves of larval gut samples. [file Image_2.tif]

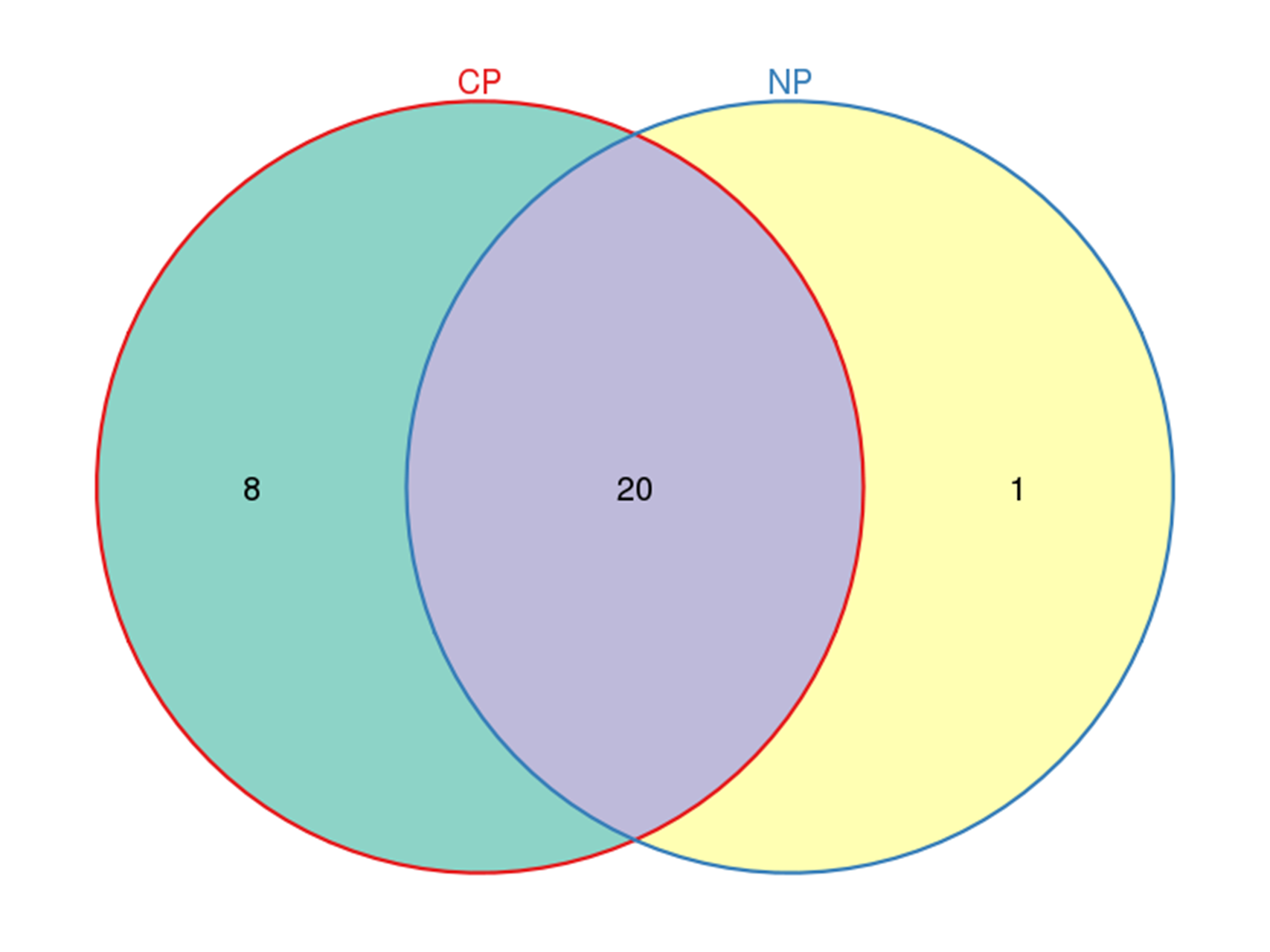

Supplement: Supplementary Figure 3 — Venn diagram of the shared and unique OTUs between parasitized larvae and non-parasitized larvae. [file Image_3.tif]

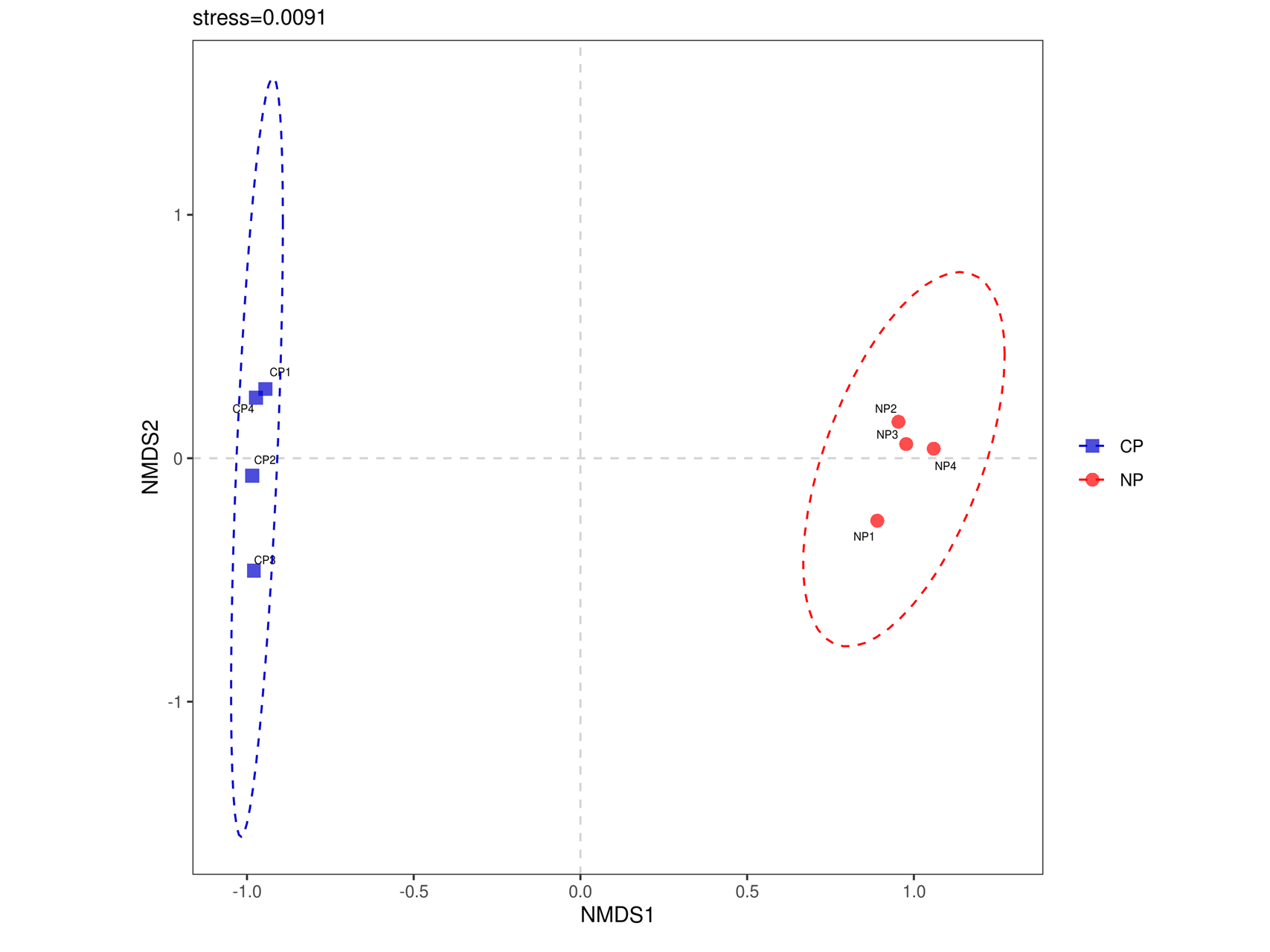

Supplement: Supplementary Figure 4 — Non-metric multidimensional scaling (NMDS) analysis. [file Image_4.tif]
