# Supplementary material for: Parasitoid Causes Cascading Effects on Plant-Induced Defenses Mediated Through the Gut Bacteria of Host Caterpillars
Source: Front Microbiol. 2021 Sep 6;12:708990. doi: 10.3389/fmicb.2021.708990 (PMC8452159; doi:10.3389/fmicb.2021.708990)
Supplement: Supplementary Table 1 — List of primers for quantitative real-time PCR for maize. [file Table_1.docx]

**Table S1 List of primers for quantitative real time PCR for maize.**

| **Gene** | **Forward** | **Reverse** | **NCBI Accession Number** | **note** |
| --- | --- | --- | --- | --- |
| ***Actin*** | GGAGCTCGAGAATGCCAAGAGCAG | GACCTCAGGGCATCTGAACCTCTC | U60511.1 | maize |
| ***MPI*** | GCGGATTATCGCCCTAACC | CGTCTGGGCGACGATGTC | X78988.2 | maize |
