# Supplementary material for: Parasitoid Causes Cascading Effects on Plant-Induced Defenses Mediated Through the Gut Bacteria of Host Caterpillars
Source: Front Microbiol. 2021 Sep 6;12:708990. doi: 10.3389/fmicb.2021.708990 (PMC8452159; doi:10.3389/fmicb.2021.708990)
Supplement: Supplementary Table 2 — OTU and sequence number in individual sample. [file Table_2.docx]

| **Table S2** OTU and sequence number in individual sample | | |
| --- | --- | --- |
| ID | OTU_Num | Seqs_Num |
| CP01 | 23 | 43582 |
| CP02 | 19 | 47907 |
| CP03 | 23 | 71587 |
| CP04 | 19 | 33373 |
| NP01 | 13 | 49218 |
| NP02 | 16 | 55469 |
| NP03 | 14 | 50332 |
| NP04 | 17 | 60874 |
| Total | 29 | 412342 |
