# Supplementary material for: Parasitoid Causes Cascading Effects on Plant-Induced Defenses Mediated Through the Gut Bacteria of Host Caterpillars
Source: Front Microbiol. 2021 Sep 6;12:708990. doi: 10.3389/fmicb.2021.708990 (PMC8452159; doi:10.3389/fmicb.2021.708990)
Supplement: Supplementary Data Sheet 1 — R script for ANOSIM and PERMANOVA. [file Data_Sheet_1.docx]

# Install R package devtools

if (!requireNamespace("BiocManager", quietly=TRUE))

install.packages("BiocManager")

suppressWarnings(suppressMessages(library(BiocManager)))

if (!requireNamespace("phyloseq", quietly=TRUE))

BiocManager::install("phyloseq")

library(phyloseq)

result=BetaDiv(otu=otutab_rare, map=metadata, group="Group",

dist="bray", method="NMDS", Micromet="adonis")

# Install R package devtools

if (!requireNamespace("devtools", quietly=TRUE))

install.packages("devtools")

library(devtools)

if (!requireNamespace("amplicon", quietly=TRUE))

install_github("microbiota/amplicon")

suppressWarnings(suppressMessages(library(amplicon)))

# Principal coordinate analysis, PCoA

(p=beta_pcoa(beta_bray_curtis, metadata, "Group"))

ggsave(paste0("p1.PCoA.bray.pdf"), p, width=89, height=56, units="mm")
