# Supplementary material for: Parasitoid Causes Cascading Effects on Plant-Induced Defenses Mediated Through the Gut Bacteria of Host Caterpillars
Source: Front Microbiol. 2021 Sep 6;12:708990. doi: 10.3389/fmicb.2021.708990 (PMC8452159; doi:10.3389/fmicb.2021.708990)
Supplement: Supplementary Data Sheet 2 — Preparation of both wheat germ diet and pinto bean diet. [file Data_Sheet_2.docx]

**Preparation of both wheat germ diet and pinto bean diet**

**Wheat germ diet**

Make up dry ingredients (do not add antibiotics)

66 g casein,

66 g dextrose

56 g wheat germ

18 g Wesson salt mix

10 g ascorbic acid

9.4 g cellulose

9.4 g sodium alginate

3.6 g cholesterol

3 g methylparaben

1 g sorbic acid

2.2 g choline chloride

Steps:

1. In a 3 L mixing bowl, combine 52 g agar and 800 mL of distilled water.
2. Heat the agar in microwave for 5 min, and then stir. Repeat for two more times to get a total of 15 min heating.
3. Meanwhile, in a 4 L mixing container, combine 1000 mL of distilled water, 10 mL wheat germ oil, 9.4 mL KOH (4M) and a bag of dry ingredients for 2 L diet.
4. Add dissolved agar and blend briefly with stick mixer. Pour immediately into sterile plastic containers with lids. Cool down the diets, and then store diets at 4℃ until use.

**Pinto bean diet**

Make up dry ingredients (do not add antibiotics)

300 g Pinto bean, ground

64 g Brewer’s yeast (fortified)

10 g Vitamin mix

6 g Ascorbic acid

4 g Methyl paraben

2 g Sorbic acid

1.5 g Propionic acid, Na salt

Steps:

1. All dry substances above were mixed and transferred into a plastic bag, which is kept at 4 ℃ before use.
2. To prepare the sterile pinto bean diet, 46 g dry ingredients stored in plastic bag were mixed with 3.2 g agar and 200 mL distilled water in an Erlenmeyer flask (500 mL).
3. The flask with diet ingredients was autoclaved at 120 ℃ for 20 min.
4. Diet was mixed evenly by a sterile spoon and then poured into 90 mm plastic petri dish (~20 mL per dish) after cooled down to ~50 ℃. All procedures were performed in a laminar flow hood.
5. Petri dishes with autoclaved diet were cooled down in laminar flow hood for at least 1 h and then stored in 4 ℃ before use.
